# Potential Impacts of Climatic Change on European Breeding Birds

**DOI:** 10.1371/journal.pone.0001439

**Published:** 2008-01-16

**Authors:** Brian Huntley, Yvonne C. Collingham, Stephen G. Willis, Rhys E. Green

**Affiliations:** 1 Institute of Ecosystem Science, School of Biological and Biomedical Sciences, Durham University, Durham, United Kingdom; 2 Conservation Science Group, Department of Zoology, University of Cambridge, Cambridge, United Kingdom; 3 Conservation Science Department, Royal Society for the Protection of Birds, Sandy, United Kingdom; Centre National de la Recherche Scientifique, France

## Abstract

**Background:**

Climatic change is expected to lead to changes in species' geographical ranges. Adaptation strategies for biodiversity conservation require quantitative estimates of the magnitude, direction and rates of these potential changes. Such estimates are of greatest value when they are made for large ensembles of species and for extensive (sub-continental or continental) regions.

**Methodology/Principal Findings:**

For six climate scenarios for 2070–99 changes have been estimated for 431 European breeding bird species using models relating species' distributions in Europe to climate. Mean range centroid potentially shifted 258–882 km in a direction between 341° (NNW) and 45° (NE), depending upon the climate scenario considered. Potential future range extent averaged 72–89% of the present range, and overlapped the present range by an average of 31–53% of the extent of the present range. Even if potential range changes were realised, the average number of species breeding per 50×50 km grid square would decrease by 6·8–23·2%. Many species endemic or near-endemic to Europe have little or no overlap between their present and potential future ranges; such species face an enhanced extinction risk as a consequence of climatic change.

**Conclusions/Significance:**

Although many human activities exert pressures upon wildlife, the magnitude of the potential impacts estimated for European breeding birds emphasises the importance of climatic change. The development of adaptation strategies for biodiversity conservation in the face of climatic change is an urgent need; such strategies must take into account quantitative evidence of potential climatic change impacts such as is presented here.

## Introduction

The response to climatic change of species' distribution and abundance patterns arises from a combination of behavioural [1], genetic [2], [3] and spatial [4] responses. However, evidence of species' responses to past climatic changes [5] leads to the expectation that the last of these responses – change in the form and/or location of the geographical range – will be of the greatest importance in relation to the rapid large-magnitude climatic changes predicted for the coming century [3]. In order to inform the development of adaptation strategies for biodiversity conservation, as well as to assess the potential consequences for ecosystem structure and function, quantitative estimates are required of the likely magnitude, direction and rates of these potential range changes. Such estimates can be made using models that relate species' distributions to present climate. To avoid reaching potentially spurious conclusions, however, estimates should be made as ‘ensemble forecasts’ obtained by modelling the potential future geographical ranges of a large number of species, preferably all of the members of one or more major taxonomic groups, under a variety of projected future climate scenarios. Because it is at extensive spatial scales that climate becomes more important than other factors, such as habitat availability, in determining species' patterns of occurrence, species' distributions also should be modelled at the scale of sub-continental or continental regions.

In order to provide such a systematic, continent-wide assessment and quantification of the potential impacts of climatic change upon all species in a taxonomic group, we have applied a consistent approach to model the relationships between breeding distribution and climate for bird species breeding in Europe. Data recording breeding distributions of birds in Europe, as presence or absence in the cells of a *ca*. 50×50 km grid, were obtained from the European Bird Census Council [6] and mean monthly climatic data for 1961–90 (‘present’) from a global dataset at 0·5°×0·5° resolution [7]. Response surface models [8] were fitted relating each species' distribution to three bioclimate variables, i.e. variables derived from the climatic data and known to be relevant to species' survival and/or performance. Robustness of the models was evaluated using a jack-knife approach, model performance being assessed using the area under the curve (AUC) of a receiver operating characteristic (ROC) plot [9]. Six climate scenarios for 2070–99 were developed from transient simulations made using three general circulation models (GCMs) for the IPCC SRES A2 and B2 emissions scenarios [10]. Potential breeding range of each species for each future climate scenario was simulated using the response surface model. Our results are based upon 431 species for which useful models could be fitted (from a total of 496 species whose European breeding distributions have been mapped [6]; see Supplementary Material (Table S1) for a list of the 431 species included in the synthesis, and Materials and Methods for the criteria for selection of these species).

Centroids of potential future and simulated present ranges (the point about which the sum of the distances of all the grid squares in which the species is simulated as present is zero, i.e. the ‘centre of gravity’ or centre of mass of the species' simulated distribution; when calculating the sum, distances to the east and north are considered positive, those west and south negative), the geodesic distance (the length of the shortest path across the Earth's surface connecting two points) between and initial azimuth (the bearing relative to north (0°) at which the geodesic path departs from the initial point) from present to future centroid, the extent and overlap of potential future ranges relative to present ranges, and the magnitude and rate of range boundary shifts necessary to realise potential range changes were evaluated for each species. Using the results, we estimated the mean distance and direction of the potential spatial displacement of species' ranges, and also the mean rate of range boundary shift required to realise these potential range changes. We also estimated the mean potential change in range extent and the mean degree of overlap between present and potential future ranges. Because the extent to which species will achieve range changes depends upon the rate of climatic change relative to the rate at which species can extend their range boundaries [11], [12], potential future number of species per grid cell was estimated for ‘worst case’ and ‘best case’ alternatives [13], i.e. assuming either that species fail to disperse, persisting only where their potential future and present ranges overlap, or that species fully realise their potential future ranges. For species whose geographical ranges are restricted or almost restricted to Europe, we also explored the extent to which they may face an increased risk of future extinction as a consequence of climatic change.

The results provide quantitative evidence of the potential impacts of climatic change on European birds, but also have general relevance in relation to impacts of climatic change on European biodiversity as a whole. Such quantitative evidence can be used to inform the development of adaptation strategies for biodiversity conservation.

## Materials and Methods

Data recording the breeding distribution of birds in Europe were obtained from the European Bird Census Council (EBCC) [6]. These data record the occurrence of breeding by each species in the *ca*. 50×50 km squares of a Universal Transverse Mercator (UTM) grid, largely during the late 1980s. The data also distinguish between grid squares where evidence of breeding by a species was sought, but not obtained, and grid squares where such evidence was not sought. In our modelling, only the first of these were used as absences, the second category being considered as missing data and excluded from model fitting for that species. Data were available for all 496 species mapped by the EBCC. These include 17 introduced species, as well as a number of native species recorded from very small numbers of grid cells. An attempt was made to fit models for 453 of these species, including 10 introduced species; no attempt was made to fit models for species with very few (<5) recorded occurrences or very sparsely scattered occurrences. For the synthesis we included the 430 native species, of the 443 that we attempted to model, that gave at least useful models, but excluded all but one of the introduced species; we included *Phasianus colchicus* (Pheasant), however, because it was introduced to many parts of Europe at least several centuries ago and is likely to have extended its range throughout most or all of the climatically suitable areas of the continent.

Mean monthly temperature, precipitation and cloudiness data for 1961–90 (‘present’) were interpolated for the mid point of each UTM grid cell from a 0·5×0·5° longitude×latitude global dataset [7]. Using previously published methods [14], values for a variety of bioclimatic variables were calculated from these monthly meteorological data. Models were fitted using combinations of a range of variables representing each of the three principal climatic constraints upon the distributions of organisms in Europe, i.e. winter cold, growing season warmth and moisture availability. For the synthesis presented here we wished to ensure consistency of modelling approach; the three variables that most often gave the best fitting model across all species were therefore used to model all species. The resultant decrease in model fit for those species best fitted by a different variable combination was marginal in all cases. The three variables used were: mean temperature of the coldest month – as a measure of winter cold; annual temperature sum above 5°C – as a measure of thermal energy available during the ‘growing season’; and an estimate of the ratio of actual to potential evapotranspiration (Priestley-Taylor α) – as a measure of the extent of annual moisture deficiency. The latter variable was estimated using a ‘bucket model’ that takes as inputs daily values of precipitation, temperature and insolation; the first two were derived from the monthly mean values for the variables, whilst the latter was estimated from the latitude of the grid cell mid-point, the day of the year and the monthly mean cloudiness [15]. It should be noted that, in using these three variables, it is not assumed that they necessarily act upon the bird species directly, indeed in many cases it is clear that the causal paths [16] through which they operate must be indirect. Winter cold, for example, cannot have a direct effect upon migrant species that are not present in the region during that season, but nonetheless is often a strong constraint upon their distributions, probably as a result of its effect upon habitat or food availability. Similarly, it is unlikely that moisture availability acts directly upon many bird species, but by acting to determine the character of the vegetation it will in turn determine availability of habitats and suitable food items.

The modelling approach used was that of fitting species–climate response surfaces [17]. For each species a response surface was fitted to the three selected bioclimatic variables; locally-weighted regression was used to fit the response surface [18]. This approach was preferred for several reasons to the many alternatives, including the multiple model and ‘model ensemble’ approaches advocated by some [19], [20]. In particular: the user makes an *a priori* choice of the variables to be included, rather than the modelling method selecting variables from some larger set input by the user; no assumption is made about the general form of the relationship between a species' probability of occurrence and a bioclimatic variable; interactions between variables are readily accommodated and, because the fitting is local, can vary across the climatic domain as evidence from autecological studies indicates is often the case [21]–[24]. Any extrapolation of the fitted model is also made very conservatively, the species' probability of occurrence beyond the margin of the fitted response surface being rapidly asymptotic to the value at the margin of that surface. Because our aim was an overall synthesis using a large ensemble of species, rather than a focus upon individual species, the uncertainties arising with respect to individual species as a result of differences in future range predictions made by models fitted using different modelling approaches also are of minimal importance. Furthermore, in at least one case where such differences have been reported [25], modelling was carried out at a spatial scale (1′×1′, i.e. *ca*. 2×2 km) much finer than that which we are considering, and at which it is very likely that factors other than climate are playing a substantial role in determining the species' pattern of distribution.

Models were fitted using all of the data (‘full’ models), and also using a very stringent jack-knife approach in which the data from all of the grid squares of each in turn of the 6° longitude×8° latitude ‘panels’ of the UTM grid system were excluded and the model fitted to the remaining data was then used to predict the species' occurrence in the omitted cells. Performance of both the ‘full’ and jack-knife models was assessed using the area under the curve (AUC) of a receiver operating characteristic (ROC) plot [9]. In order to express the probabilities of occurrence simulated by the models as simulations of presence or absence of the species, the threshold probability that maximised Cohen's κ [26] was determined [8], [14]. Although it has been suggested, on the basis of tests with small artificial datasets (100 samples), that this is not the best approach for determining thresholds [27], the results of that comparative study nonetheless led its authors to conclude that “*the best result will probably be obtained by any approach*” [27, p. 392]; furthermore, we are aware of no systematic study of alternative methods that has been performed for large datasets and across many species.

The AUC values for the ‘full’ and jack-knife models for all 453 species for which we attempted to fit models are illustrated in the Supplementary Material (Figure S1). Almost 89% of the ‘full’ models fitted gave AUC values >0·9, indicating a ‘high’ model performance [28], whereas <2% of the models gave AUC values ≤0·7. Of the jack-knife models, although only 28% gave AUC values >0·9, a further 60% gave AUC values >0·7, indicating that they were ‘useful’. The jack-knife model performance was most markedly reduced, compared to that of the ‘full’ model, for species with geographically restricted distributions; in such cases leaving out a ‘panel’ of grid cells often resulted in omitting a substantial proportion of the species' recorded presences. The synthesis presented here is based upon the ‘full’ models and includes the 430 native species that gave models with a ‘useful’ or ‘high’ performance and one introduced species (*Phasianus colchicus* – Pheasant), the model for which had a ‘high’ performance.

The six potential future climate scenarios used were for the period 2070–99 and were developed from transient simulations made using three of the general circulation models (GCMs) included in the IPCC 2001 synthesis [29] and for two of the IPCC SRES emissions scenarios, the A2 and B2 scenarios [10]. All three GCMs selected, GFDL_R30_c [30] (GFDL), HadCM3 [31] and ECHAM4/OPYC3 [32] (ECHAM4), have an equilibrium sensitivity for global mean temperature close to the mean for the nine models included in the IPCC synthesis [29]. With respect to their simulated precipitation, however, GFDL is relatively ‘wet’, the HadCM3 model is close to the mean for all nine models, and the ECHAM4 model is relatively ‘dry’. The A2 emissions scenario is one of continuing relatively rapid population growth and relatively high emissions, global CO_2_ emissions increasing to almost 5× 1990 values by 2100, whereas the B2 scenario is one of slower but continuing population growth and diverse technological change resulting in global CO_2_ emissions just over double their 1990 values by 2100. In each case the future climate scenario used to model the potential impacts was obtained by calculating the anomalies between the GCM simulated mean monthly temperature (additive anomaly) and precipitation (multiplicative anomaly) for 2070–99 and 1961–90 for the GCM grid cells, interpolating these to the UTM grid, applying them to the values interpolated for the UTM grid from the 1961–90 observed data, and finally calculating values for the three bioclimate variables.

The potential breeding range of each species for each future climate scenario was simulated using the species–climate response surface. Because this modelling approach does not attempt to simulate either the species' population dynamics or the dispersal of offspring, the two principal components of the process by which species' range shifts are achieved, the simulated range is not a ‘projection’; what is simulated is the species' ‘potential’ future range, i.e. the range it could occupy if it fully achieved the range shift necessary to occupy all of those areas with future climatic conditions equivalent to those in some part of its present range. In order to reduce any effects of model bias, future range extent (*R*) and overlap (*O*) were calculated relative to the species' range simulated in the same way for the present climate. The centroids of the potential future and simulated present ranges were calculated; geodesic distance (*D* – km) and initial azimuth (*θ*) from the present to the future centroid were computed using a Fortran program implementing the solution of Sodano and Robinson [33].

For each species and scenario, the range boundary shift necessary to realise the potential range displacement was estimated as the 90^th^ percentile of the distribution of geodesic distance between each grid cell simulated as newly suitable and the nearest grid cell simulated as suitable at the present. Given that this shift would need to be realised during the interval between 1961–90 and 2070–99, i.e. within 109 years, the rate of range boundary shift (*V* – km yr^−1^) was estimated by dividing the estimated range boundary shift by 109.

Estimates of the potential relative number of species per grid cell were made using only those grid cells for which presence or absence could be simulated for at least 395 of the species considered. Estimates were made for the ‘worst case’ (*N*′) and ‘best case’ (*N*) alternatives, i.e. assuming either that species failed to disperse and persisted only in the area of overlap between the potential future and present ranges or that species fully realised their potential future ranges.

## Results

### Range shifts

**Figure 1:**
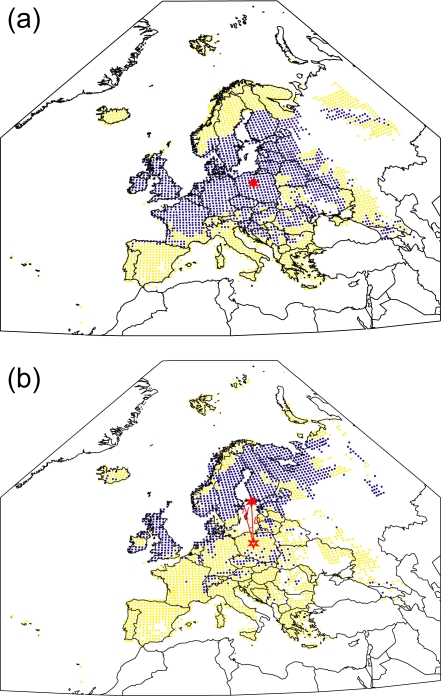
Simulated present and potential future ranges of *Locustella naevia.* (a) Simulated distribution of *Locustella naevia* (Grasshopper Warbler) in Europe for the ‘present’ (1961–90) climate. Blue symbols represent grid squares simulated as suitable, yellow symbols grid squares simulated as unsuitable, and white areas of the map regions with climatic conditions unlike those anywhere where data for the species are available in the EBCC dataset [6]. The red star indicates the position of the centroid of the species' simulated range. The response surface model has a ‘high’ performance (AUC = 0·952) as assessed by its ability to describe the observed distribution as recorded in the EBCC atlas [6]. (b) Simulated potential distribution of *Locustella naevia* in Europe for the HadCM3 B2 future climate scenario (2070–99). Blue and yellow symbols, and white areas of the map, as in (a). For this climate scenario the species' potential future range extent, in terms of potentially suitable grid squares, is 66% of the extent of its simulated present range. The area of overlap of the simulated future potential and present ranges is 37% of the extent of the latter. The red star indicates the position of the centroid of the species' simulated potential future range; the red star outline indicates the position of the centroid of the species' simulated range for the ‘present’ climate. The line joining these two stars, length *D* (808 km), represents the geodesic path between the two centroids. Also plotted is a line indicating the direction of north (N) from the centroid of the species' simulated range for the ‘present’ climate; the angle *θ* (10·6°) between north and the geodesic path between the two centroids is the initial azimuth of the geodesic path.

**Figure 2:**
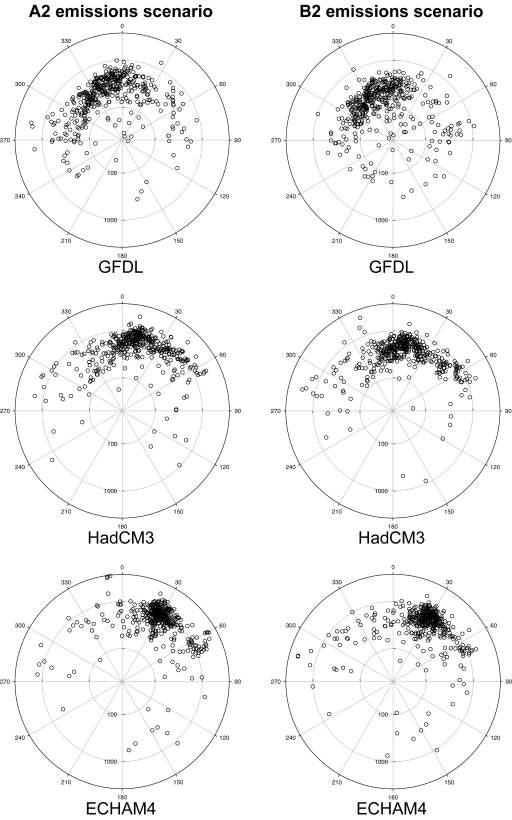
Polar plots of potential range centroid displacement. The distance and direction of each species' potential range centroid displacement (see Figure 1) is illustrated for the six potential future climate scenarios, each point in each plot representing an individual species. The radial distance from the centre of the plot at which a point is located indicates the geodesic distance (*D*) between the centroids of its simulated present and potential future ranges; the angular position of a point indicates the initial azimuth (*θ*) of the geodesic path between the present and potential future range centroids as a bearing relative to North (0°). The same logarithmic scale is used to plot radial distances in all six plots; the centre point of each plot represents a geodesic distance of 20 km, whilst their circumferences correspond to a distance of 3750 km.

Figure 1 illustrates the simulated ‘present’ and potential future (HadCM3 B2 scenario) distributions of *Locustella naevia* (Grasshopper Warbler). This species is selected for this purpose because it is in many senses close to the ‘average’ of the 431 species in the ensemble. The centroids of the two simulated ranges are shown, as are the geodesic distance (*D*) between the two centroids, and the initial azimuth (*θ*) of the geodesic path from the centroid of the ‘present’ range to that of the potential future range. Figure 2 shows, using polar plots, the magnitudes and directions of the potential range centroid shifts for all 431 species for each of the six future climate scenarios.

For each future climate scenario, Table 1 presents the mean and range of the geodesic distances (*D*) and the mean initial azimuth (*θ*) for all species. The mean geodesic distance ranged from 258 to 882 km, the range for individual species and across all future scenarios being much greater (20–3578 km). The mean initial azimuth ranged from 341° (NNW) to 45° (NE), although as Figure 2 illustrates this too varied considerably amongst species. Table 1 also presents the mean and range of the rates of range boundary extension (*V*) required to realise potential range changes for each of the six scenarios. The mean rate ranged from 3·61 to 6·62 km yr^−1^, with extreme values for individual species and scenarios of 0·37 and 24·85 km yr^−1^. Several types of evidence can be used to estimate rates at which species are likely to achieve extensions of their range boundaries. Historical distribution data allow species' range boundary changes of the past few decades, interpreted as responses to recent climatic change, to be estimated. In a global study, average rates of 0·61±0·24 km yr^−1^ were reported across a range of taxa [4], although a more recent study in the United Kingdom that examined a wider range of taxonomic groups reported faster mean rates of range extension, especially for less well recorded grid squares, of 1·37 to 2·48 km yr^−1^ [34]. Despite the relative rapidity of these mean range extensions, there is evidence that many species have not fully realised potential range boundary extensions because other factors, such as habitat loss and fragmentation, are acting to limit both their population size and the effectiveness of their dispersal [12]. Palaeoecological evidence from the late Quaternary shows that terrestrial organisms from many taxonomic groups extended their ranges in response to the rapid climatic changes at the transition from the last glacial stage to the Holocene, about 11,500 years ago. These data can also be used to estimate rates of range boundary extension, although systematic estimates are restricted principally to higher plants [35], [36]. Rates of 0·2 to 2·0 km yr^−1^ have been reported for both Europe [35] and eastern North America [36], although recent molecular genetic evidence suggests that for at least some taxa in the latter region these rates are likely to be over-estimates [37]. Although molecular genetic evidence [38], [39] shows that birds exhibited similar magnitudes of late-Quaternary range extension, too few records of avian fossils are available to allow direct estimates of the rates at which their range boundary extensions were achieved. A third line of evidence relates to range boundary extensions achieved by invasive introduced species and by the very few species that have extended their natural range by colonising previously unoccupied geographical regions during historical times (e.g. *Streptopelia decaocto* (Collared Dove) that has expanded across Europe from Turkey since *ca*. 1900). Although the mean rate of 43·2 km yr^−1^ reported for seven studies of such species [40] is much more rapid than mean rates estimated from other lines of evidence, this is not unexpected given that these species are by definition characterised by their rapidly invasive behaviour. Evidence from past range changes thus indicates that most species, apart from those of an invasive character, are likely to extend their range boundaries at rates little more than half that required to realise their potential range boundary extension even for the most modest of the climatic change scenarios examined (GFDL B2). For the other scenarios examined, many species' range boundary extension rates are likely to be at least an order of magnitude less than those required to realise potential range changes. It thus must be expected that many species will fail to realise fully their potential future ranges within the present century. Although a small minority of native species may prove to have the characteristics required for invasive behaviour, and thus fully realise their potential range changes, it is not possible on the basis of our present knowledge and understanding to predict with any confidence which they may be.

**Table 1:** Summary of potential impacts

	A2 emissions scenario			B2 emissions scenario		
	GFDL	HadCM3	ECHAM4	GFDL	HadCM3	ECHAM4
*D* (km)	361	707	882	258	545	680
	25–1825	20–2153	89–3578	25–1342	54–2477	58–2436
*θ* (° from N)	342·8°	11·7°	45·0°	341·3°	9·3°	27·8°
*V* (km yr^−1^)	4·41	6·55	6·62	3·61	5·14	5·87
	0·46–21·72	0·43–23·06	0·37–24·85	0·44–22·13	0·46–24·26	0·37–23·05
*R*	0·722	0·757	0·892	0·812	0·805	0·803
*O*	0·429	0·312	0·475	0·528	0·394	0·375
*R_0_*	5	8	22	4	6	21
*R_10_*	1	22	25	1	8	9
*O_0_*	18	54	59	13	27	43
*O_10_*	27	65	78	14	51	60
*N*	0·874	0·848	0·768	0·932	0·914	0·858
*N*′	0·657 (2881)	0·516 (3074)	0·436 (3550)	0·733 (3067)	0·602 (3145)	0·520 (3521)

*D* geodesic distance (mean, minimum and maximum) between centroids of range simulated for present climate and of potential range simulated for future climate scenario

*θ* mean bearing of initial azimuth for geodesic path between centroids of range simulated for present climate and of potential range simulated for future climate scenario

*V* rate of range boundary adjustment (mean, minimum and maximum) necessary to achieve potential range displacement

*R* mean extent of potential future range, measured as number of potentially occupied grid cells, expressed as a proportion of simulated present range

*O* extent of overlap between potential future range and simulated present range, expressed as a proportion of simulated present range extent

*R_0_* number of species with zero potential future range extent in Europe

*R_10_* number of species with a non-zero potential future range extent in Europe that is less than one tenth of the extent of their simulated present range

*O_0_* number of species with zero overlap between potential future range and simulated present range

*O_10_* number of species with a non-zero overlap between their potential future range and simulated present range that is less than one tenth of the extent of the latter

*N* mean potential future species number per grid cell as a proportion of mean present species number per grid cell, assuming ‘perfect’ dispersal

*N*′ mean potential future species number per grid cell as a proportion of mean present species number per grid cell, assuming ‘dispersal failure’; (number of grid cells used to calculate N and N′ is shown in parentheses)

### Range extents

In those cases where species do not realise fully their potential range extension, the magnitude of the climatic change relative to the breadth of the species' climatic niche becomes important, determining the extent of the overlap between the species' future potential and realised ranges. Species for which this overlap becomes small relative to their initial range are most likely to suffer associated population reduction and may have an increased risk of extinction. Species whose future potential range is markedly smaller in extent than their present range also face the likelihood of population reduction and heightened extinction risk [13], even if they are able to respond sufficiently rapidly that their realised range corresponds to their potential range. Mean overlap (*O*) and extent (*R*) of species' potential future ranges relative to their present ranges are presented in Table 1, as are the numbers of species with potential future overlaps (*O_0_*) or range extents (*R_0_*) of zero or of less than one tenth (*O_10_*, *R_10_*) the extent of their present ranges. Regardless of the scenario, mean overlap rarely exceeded *ca*. 50% and mean future range extent *ca*. 80% of the extent of the present range. Similarly, even the most moderate scenario resulted in some species with zero overlap or zero potential future range extent in Europe; in the most extreme scenarios *ca*. 14% and *ca*. 5% of species respectively were in these two categories. For most scenarios a larger number of species had future overlaps or range extents that are non-zero but <10% of the extent of their present range.

### Species-richness

The simulated general reduction in mean range extent resulted in a reduction in the simulated mean number of breeding species per grid cell (Table 1), and hence a general decrease in local avian species-richness across Europe. As in previous studies of the potential impacts of climatic change [13], this number was calculated in two ways, firstly assuming ‘perfect’ dispersal (*N*), i.e. species fully realise their potential range as simulated for a future climate scenario, and secondly assuming ‘dispersal failure’ (*N*′), i.e. species occupy none of the newly climatically suitable areas but fail to persist in parts of their present range that are no longer climatically suitable. The smallest impact amongst the scenarios examined, for the GFDL B2 scenario and assuming ‘perfect’ dispersal, was a 6·8% average reduction in species number breeding in a 50 km grid square, whereas the largest impact, for the ECHAM4 A2 scenario and an assumption of ‘dispersal failure’, was a 56·4% reduction.

### Extinction risk

The impacts of limited overlap and/or markedly reduced range extent are of particular conservation significance in the case of species that either are endemic to our study area or nearly so (≥90% of their overall breeding range/population in Europe). Table 2 lists the 10 endemic and 30 near-endemic species for which useful models could be fitted, and presents the overlap (*O*) and relative extent (*R*) of their potential future ranges for the six future climate scenarios. Most of these species had relatively limited overlap; a substantial minority (22·5%) had zero overlap for one or more of the scenarios. There was, however, highly significant variation in overlap among the GCM x emissions scenario combinations (effect of GCM/emissions scenario in a two-way ANOVA of logit-transformed values with species as the other main effect: *F*_5,195_ = 19·75, *p*<0·001). For a given species, overlap tended to be lowest for ECHAM4 and highest for GFDL, with HadCM3 intermediate or similar to ECHAM4; overlap tended to be higher for the B2 than the A2 emissions scenario. The relative extent of the potential future range was much more varied; some species showed substantial decreases whereas others showed marked increases. The latter tendency was especially common amongst species with present ranges restricted to southern Europe. Relative future range extent did not vary significantly among the six GCM x emissions scenario combinations (effect of GCM/emissions scenario in a two-way ANOVA of log-transformed values with species as the other main effect: *F*_5,195_ = 1·78, *p* = 0·14).

**Table 2:** Present and potential future ranges of endemic and near-endemic species

Species	Status	Range extent (no. of grid cells)	Potential future range extent (*R*) and overlap (*O*) (proportion of extent of simulated present range; upper figures are for the A2 and lower for the B2 emissions scenario)					
		observed/simulated	GFDL		HadCM3		ECHAM4	
			*R*	*O*	*R*	*O*	*R*	*O*
*Calonectris diomedea*	NE/m	80/109	0·394/0·055		0·596/0·083		0·394/0·092	
Cory's Shearwater			0·294/0·138		0·495/0·055		0·541/0·055	
*Puffinus yelkouan*	NE/pm	68/90	0·211/0·011		0·300/0·000		0·167/0·000	
Yelkouan Shearwater			0·278/0·100		0·356/0·000		0·200/0·000	
*Phalacrocorax aristotelis*	NE	283/290	0·976/0·500		0·797/0·479		0·859/0·386	
Shag			1·069/0·555		0·841/0·441		0·931/0·466	
*Milvus milvus*	NE/pm	632/659	0·599/0·259		0·419/0·035		0·624/0·041	
Red Kite			0·756/0·393		0·580/0·138		0·624/0·103	
*Accipiter brevipes*	NE/m	107/146	0·411/0·130		0·767/0·192		0·247/0·151	
Levant Sparrowhawk			0·610/0·281		1·144/0·288		0·658/0·267	
*Aquila adalberti*	E	40/46	2·065/0·435		2·370/0·000		2·478/0·000	
Spanish Imperial Eagle			2·000/0·370		1·652/0·000		2·196/0·000	
*Falco eleonorae*	NE/m	55/81	1·086/0·247		4·741/0·370		2·679/0·210	
Eleonora's Falcon			0·975/0·358		2·938/0·358		2·136/0·235	
*Alectoris graeca*	E	235/255	1·682/0·161		2·039/0·129		2·639/0·059	
Rock Partridge			1·298/0·286		1·949/0·161		2·247/0·098	
*Alectoris rufa*	E	570/611	0·753/0·462		0·822/0·278		0·830/0·291	
Red-legged Partridge			1·020/0·635		0·809/0·347		0·825/0·326	
*Porzana parva*	NE/m	382/615	0·600/0·276		0·610/0·133		0·400/0·024	
Little Crake			0·743/0·302		0·654/0·213		0·528/0·140	
*Stercorarius skua*	E/m	67/61	0·475/0·213		0·262/0·115		0·541/0·230	
Great Skua			0·770/0·361		0·639/0·180		0·672/0·279	
*Larus melanocephalus*	NE/pm	102/121	0·099/0·000		0·653/0·025		0·364/0·000	
Mediterranean Gull			0·174/0·008		0·719/0·066		0·413/0·008	
*Larus audouinii*	NE/pm	39/51	0·137/0·000		0·255/0·000		0·176/0·000	
Audouin's Gull			0·412/0·157		0·137/0·000		0·059/0·000	
*Picus viridis*	NE	1754/2057	0·868/0·687		0·943/0·557		1·027/0·514	
Green Woodpecker			0·974/0·778		0·996/0·661		1·092/0·667	
*Dendrocopos medius*	NE	889/1165	0·758/0·415		0·647/0·130		0·707/0·022	
Middle Spotted Woodpecker			0·877/0·524		0·825/0·336		0·775/0·144	
*Lullula arborea*	NE/pm	1698/2079	0·702/0·554		0·902/0·528		0·965/0·506	
Woodlark			0·835/0·702		0·941/0·601		0·999/0·578	
*Anthus pratensis*	NE/pm	1620/2268	0·456/0·432		0·448/0·403		0·496/0·420	
Meadow Pipit			0·567/0·541		0·555/0·510		0·584/0·505	
*Anthus petrosus*	E/pm	268/308	0·744/0·545		0·740/0·536		0·666/0·468	
Rock Pipit			0·744/0·552		0·818/0·588		0·740/0·539	
*Prunella modularis*	NE/pm	2004/2672	0·699/0·658		0·601/0·549		0·673/0·639	
Dunnock			0·784/0·747		0·719/0·665		0·763/0·721	
*Erithacus rubecula*	NE/pm	2569/3254	0·757/0·716		0·738/0·680		0·823/0·760	
Robin			0·828/0·787		0·815/0·754		0·892/0·829	
*Saxicola rubetra*	NE/m	2148/2866	0·654/0·613		0·523/0·477		0·604/0·558	
Whinchat			0·778/0·736		0·693/0·645		0·673/0·629	
*Turdus torquatus*	NE/pm	544/567	1·166/0·310		0·935/0·293		1·146/0·312	
Ring Ouzel			1·222/0·388		1·079/0·360		1·145/0·370	
*Acrocephalus paludicola*	NE/m	61/88	0·670/0·011		0·739/0·000		0·443/0·000	
Aquatic Warbler			1·023/0·011		0·784/0·000		0·557/0·000	
*Acrocephalus palustris*	NE/m	1376/1945	0·796/0·663		0·746/0·445		0·828/0·448	
Marsh Warbler			0·899/0·768		0·881/0·623		0·903/0·563	
*Hippolais icterina*	NE/m	1432/2017	0·569/0·484		0·452/0·271		0·547/0·307	
Icterine Warbler			0·689/0·604		0·607/0·424		0·644/0·418	
*Sylvia balearica/ S. sarda*	E/pm	37/51	0·824/0·020		1·020/0·000		1·843/0·000	
Balearic Warbler/ Marmora's Warbler			1·157/0·294		0·608/0·000		0·961/0·000	
*Sylvia undata*	NE	459/522	1·042/0·670		1·071/0·303		1·437/0·460	
Dartford Warbler			1·159/0·784		0·864/0·385		1·266/0·490	
*Sylvia atricapilla*	NE/pm	2536/3106	0·913/0·823		0·921/0·760		1·090/0·873	
Blackcap			0·956/0·883		0·960/0·812		1·100/0·902	
*Regulus ignicapillus*	NE/pm	1048/1124	0·873/0·483		0·898/0·367		0·978/0·202	
Firecrest			0·957/0·618		0·874/0·436		0·959/0·317	
*Ficedula albicollis*	NE/m	442/629	0·909/0·402		0·653/0·021		0·700/0·021	
Collared Flycatcher			0·992/0·482		0·758/0·200		0·661/0·049	
*Parus cristatus*	NE	1590/2085	0·731/0·561		0·609/0·343		0·816/0·467	
Crested Tit			0·858/0·683		0·737/0·500		0·886/0·523	
*Parus caeruleus*	NE	2526/2950	0·905/0·816		1·019/0·817		1·149/0·893	
Blue Tit			0·940/0·858		1·048/0·868		1·153/0·905	
*Certhia brachydactyla*	NE	1226/1348	0·992/0·646		0·980/0·390		1·141/0·361	
Short-toed Treecreeper			1·054/0·760		1·056/0·567		1·136/0·448	
*Cyanopica cyanus*	E	105/134	1·164/0·410		0·978/0·000		1·470/0·060	
Azure-winged Magpie			1·209/0·493		0·754/0·045		1·164/0·090	
*Sturnus unicolor*	NE	291/324	0·806/0·485		0·528/0·065		0·843/0·142	
Spotless Starling			0·886/0·617		0·435/0·136		0·765/0·204	
*Passer×italiae*	E	185/196	1·704/0·071		2·454/0·061		2·816/0·000	
Italian Sparrow			1·372/0·148		1·995/0·143		2·367/0·005	
*Serinus citrinella*	E/pm	169/171	0·719/0·222		0·415/0·082		0·643/0·035	
Citril Finch			0·936/0·240		0·538/0·094		0·520/0·094	
*Loxia scotica*	E	14/7	0·429/0·000		2·000/0·000		2·143/0·000	
Scottish Crossbill			0·286/0·000		2·857/0·000		3·143/0·000	
*Loxia pytyopsittacus*	NE	471/781	0·494/0·384		0·373/0·251		0·531/0·287	
Parrot Crossbill			0·569/0·472		0·583/0·408		0·671/0·389	
*Emberiza cirlus*	NE	882/992	1·412/0·778		1·721/0·751		1·965/0·729	
Cirl Bunting			1·356/0·854		1·538/0·794		1·820/0·769	

Species' status is categorised as: E–endemic; NE–near-endemic; m–long-distance migrant; pm–short-distance or partial migrant. Migratory status was determined according to the predominant behaviour of the population breeding in Europe.

Range extent is expressed as the number of grid squares in which the species is observed or simulated to be present; simulated ranges are often more extensive because species were simulated for ‘no data’ grid squares that fell within the climatic space of the data.

Two species classified as endemic and one classified as near-endemic are omitted from the Table. The distribution of *Sitta whiteheadi* (E; 6 recorded occurrences) could not be modelled, whilst the models obtained for *Tetrao mlokosiewiczi* (NE; 50 recorded occurrences) and *Tetraogallus caucasicus* (E; 30 recorded occurrences) were not considered useful and were rejected.

**Figure 3:**
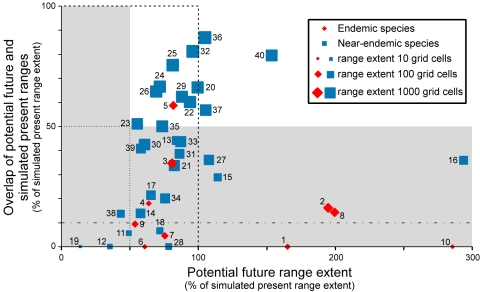
Overlap vs relative extent of potential future range. The percentage of the present simulated range that is projected to remain climatically suitable is plotted against the extent of the future potential range expressed as a percentage of the simulated present range. Each point represents one of the ten bird species endemic to Europe (red diamonds) or one of the 30 species that are nearly so (blue squares) for which models could be fitted. Numbers identify species (see Table below). Results shown are for the HadCM3 B2 scenario. The size of the symbols is proportional to the log of the simulated present range extent. Dashed lines indicate the ‘no-change’ value of 100% for overlap and relative extent. Zones with overlap and/or relative extent <50% are shaded and bounded by dotted lines. The dot-dashed line bounds the zone with overlap <10%. Key to numbers used to identify species in Figure 3 **Endemic species**        13 *Phalacrocorax aristotelis* 27 *Turdus torquatus* 1 *Aquila adalberti* 14 *Milvus milvus* 28 *Acrocephalus paludicola* 2 *Alectoris graeca* 15 *Accipiter brevipes* 29 *Acrocephalus palustris* 3 *Alectoris rufa* 16 *Falco eleonorae* 30 *Hippolais icterina* 4 *Stercorarius skua* 17 *Porzana parva* 31 *Sylvia undata* 5 *Anthus petrosus* 18 *Larus melanocephalus* 32 *Sylvia atricapilla* 6 *Sylvia balearica/S. sarda* 19 *Larus audouinii* 33 *Regulus ignicapillus* 7 *Cyanopica cyanus* 20 *Picus viridis* 34 *Ficedula albicollis* 8 *Passer × italiae* 21 *Dendrocopos medius* 35 *Parus cristatus* 9 *Serinus citrinella* 22 *Lullula arborea* 36 *Parus caeruleus* 10 *Loxia scotica* 23 *Anthus pratensis* 37 *Certhia brachydactyla* **  Near-endemic species** 24 *Prunella modularis* 38 *Sturnus unicolor* 11 *Calonectris diomedea* 25 *Erithacus rubecula* 39 *Loxia pytyopsittacus* 12 *Puffinus yelkouan* 26 *Saxicola rubetra* 40 *Emberiza cirlus*

An alternative way to view these potential impacts upon endemic and near endemic species is illustrated in Figure 3. By combining information about the extent of each species' present range with the overlap and relative extent of its potential future range, this plot highlights the observation that species with more limited present range extent are likely to have more restricted overlap (Spearman rank correlation *r_S_* = 0·867, *p*<0·001). Such species face a particular threat from climatic change because the small extent of their present range reflects a small population and thus a limited output of potential colonists of newly climatically suitable areas. They are thus likely to be amongst the species least able to achieve the necessary range adjustments to adapt to climatic change. That their future potential ranges generally also had a small overlap with their present range further heightens the risk that they may be driven to extinction by the effects of climatic change. The ten species with *O* <10% of the extent of their present range for this scenario are probably amongst those most likely to suffer negative consequences from climatic change. Given that the populations of these ten species also are between 8× and >1900× smaller (median 33·5×) than those of close relatives (Supplementary Material, Table S2), their ability to adapt to climatic change can be expected to be much more limited than that of their more widespread and populous relatives that also generally face less extreme demands for adaptation.

## Discussion

Our results show that the potential impact of climatic change on European birds is a combination of: very rapid potential range displacements of large magnitude, generally in a northward direction; an average reduction in the extent of species' ranges; a limited mean overlap between species' potential future and present ranges; a general reduction in the number of species breeding in any area; and an increased extinction risk for some species, including a number of those endemic or near endemic to Europe.

No comparable synthesis of modelling results for a large ensemble of species and at a continental scale, such as that presented here for the birds breeding in Europe, has previously been reported to our knowledge. Nonetheless, evidence from those studies that have been reported on other groups and in other regions [41]–[43] suggests that our results can serve as a general model for other groups and continents. In terms of the development of adaptation strategies, the rate and magnitude of potential distribution shifts pose perhaps the most fundamental challenges. Conservation strategies have to-date relied heavily upon the identification and designation of protected areas (PAs); as others already have argued, however, climatic change is likely to result in some species being unable to persist in those PAs where they now occur [44], [45], including in some cases perhaps the species whose conservation initially prompted the establishment of the PA. In addition, the rates of range-boundary adjustment required to track climatic change are, as we have shown, unlikely to be achieved except by a minority of species with ‘invasive’ characteristics. Furthermore, the majority of species will require relatively closely spaced habitat patches if they are to achieve even the relatively slow rates of range boundary adjustments that are more typical. Adaptation strategies thus must have at their core an extension of conservation measures and species' protection to the wider landscape. This is not to be taken as implying that PAs will be of any less value in future. Existing PAs, although they may lose some of the species for which they were designated, are likely to continue to protect important habitats from damage. Furthermore, their optimal management is likely to assist those species declining in a region because of climate change by delaying their disappearance and thus providing a continuing source of propagules/offspring for dispersal to areas with more favourable climate. For those species for which the climate of a region is becoming more suitable, existing PAs are likely to offer prime sites for colonisation and thus ‘bridgeheads’ for range extension. In these roles, among others, increases in the extent of existing PAs and the establishment of new PAs are likely to be beneficial in meeting future conservation targets, as Hannah *et al*. [45] have argued. However, whilst they will remain necessary, PAs will not of themselves be sufficient. If species are to achieve their potential range shifts then the wider landscape must provide ‘corridors’ [46] or ‘stepping stones’ *via* which species can cross the generally inhospitable matrix of heavily managed landscapes dominated by agriculture, intensive forestry and other human land-uses that are inimical to wildlife. Given the magnitude of potential distribution shifts, the individualism displayed by species with respect to the direction of their range shifts, and the inherent uncertainties in the projection both of future climatic conditions and of their impacts, ‘corridors’ will generally be a less viable option than ‘stepping stones’ because the latter, if appropriately implemented, can support range boundary shifts in any direction and of any magnitude across the landscape. Given, however, that in many parts of the world the magnitude of the range shifts not only will result in species complements of individual PAs potentially changing, but will result in species' distributions potentially shifting into countries where they are not currently present, the development of adaptation strategies must in most regions be undertaken as a collaborative international effort.

The potential reductions in the extent of species' ranges, and the consequent potential reductions in local species richness, represent a further challenge for biodiversity conservation. In this case, adaptation strategies must be designed to mitigate these potential changes as far as possible, principally through a combination of increases in the extent of PAs and the extension of species' protection measures to the wider landscape, including the ‘stepping stone’ habitat patches established therein. Projected climatic change during the present century also can be expected to increase extinction risk [13], especially for species with small range extents and those occupying climatic conditions that are unlikely to persist in the future. Identifying species at greatest risk and monitoring their distribution and population must form part of adaptation strategies, as must the implementation of management measures designed to reduce the negative impacts of climatic change where these are projected to occur. In the most extreme cases captive breeding programmes and/or deliberate translocations may offer the only hope for the most severely impacted species.

Ultimately, climatic change can be expected to cause massive re-arrangements of ecosystems comparable to those that characterised the transition from the last glacial to the post glacial [47]. Conserving biodiversity during such a period of ecological upheaval poses many new challenges and will require a considerable increase in the resources available, especially to achieve the increase in the area that is protected for wildlife that will be necessary in order to sustain species as they adjust their ranges in response to climatic change.

## Supporting Information

Table S1 Species included in the synthesis
(0.05 MB PDF)

Table S2 Population estimates for Europe for 10 endemic and near-endemic species† and for examples of their widespread relatives.
(0.02 MB PDF)

Figure S1 Distribution of AUC values for fitted models. Solid bars show values for ‘full’ models fitted to all data, open bars show values for models fitted using the jack-knife procedure.
(0.09 MB EPS)
