# Supplementary material for: Potential Impacts of Climatic Change on European Breeding Birds
Source: PLoS One. 2008 Jan 16;3(1):e1439. doi: 10.1371/journal.pone.0001439 (PMC2186378; doi:10.1371/journal.pone.0001439)
Supplement: Table S1 — Species included in the synthesis (0.05 MB PDF) [file pone.0001439.s001.pdf]

Table S1 : Species included in the synthesis

Scientific name	**Common name** (English)
Gavia stellata	Red-throated Diver
Gavia arctica	Black-throated Diver
Gavia immer	Great Northern Diver
Tachybaptus ruficollis	Little Grebe
Podiceps cristatus	Great Crested Grebe
Podiceps grisegena	Red-necked Grebe
Podiceps auritus	Slavonian Grebe
Podiceps nigricollis	Black-necked Grebe
Fulmarus glacialis	Fulmar
Calonectris diomedea	Cory’s Shearwater
Puffinus puffinus	Manx Shearwater
Puffinus yelkouan	Yelkouan Shearwater
Hydrobates pelagicus	Storm Petrel
Oceanodroma leucorhoa	Leach’s Storm-petrel
Morus bassanus	Gannet
Phalacrocorax carbo	Cormorant
Phalacrocorax aristotelis	Shag
Phalacrocorax pygmeus	Pygmy Cormorant
Botaurus stellaris	Bittern
Ixobrychus minutus	Little Bittern
Nycticorax nycticorax	Night Heron
Ardeola ralloides	Squacco Heron
Bubulcus ibis	Cattle Egret
Egretta garzetta	Little Egret
Egretta alba	Great White Egret
Ardea cinerea	Grey heron
Ardea purpurea	Purple Heron
Ciconia nigra	Black Stork
Ciconia ciconia	White Stork
Plegadis falcinellus	Glossy Ibis
Platalea leucorodia	Spoonbill
Phoenicopterus ruber	Greater Flamingo
Cygnus olor	Mute Swan
Cygnus columbianus	Bewick’s Swan
Cygnus cygnus	Whooper Swan
Anser fabalis	Bean Goose
Anser brachyrhynchus	Pink-footed Goose
Anser erythropus	Lesser White-fronted Goose
Anser anser	Greylag Goose
Branta leucopsis	Barnacle Goose
Branta bernicla	Brent Goose
Tadorna ferruginea	Ruddy Shelduck
Tadorna tadorna	Shelduck
Anas penelope	Wigeon
Anas strepera	Gadwall
Anas crecca	Teal
Anas platyrhynchos	Mallard
Anas acuta	Pintail
Anas querquedula	Garganey
Anas clypeata	Shoveler
Marmaronetta angustirostris	Marbled Teal
Netta rufina	Red-crested Pochard
Aythya ferina	Pochard
Aythya nyroca	Ferruginous Duck
Aythya fuligula	Tufted Duck

Table S1 (continued)

Scientific name	**Common name** (English)
Aythya marila	Scaup
Somateria mollissima	Eider
Somateria spectabilis	King Eider
Histrionicus histrionicus	Harlequin Duck
Clangula hyemalis	Long-tailed Duck
Melanitta nigra	Common Scoter
Melanitta fusca	Velvet Scoter
Bucephala clangula	Goldeneye
Mergus albellus	Smew
Mergus serrator	Red-breasted Merganser
Mergus merganser	Goosander
Oxyura leucocephala	White-headed Duck
Pernis apivorus	Honey Buzzard
Elanus caeruleus	Black-winged Kite
Milvus migrans	Black Kite
Milvus milvus	Red Kite
Haliaeetus albicilla	White-tailed Eagle
Gypaetus barbatus	Lammergeier
Neophron percnopterus	Egyptian Vulture
Gyps fulvus	Griffon Vulture
Aegypius monachus	Black Vulture
Circaetus gallicus	Short-toed Eagle
Circus aeruginosus	Marsh Harrier
Circus cyaneus	Hen Harrier
Circus macrourus	Pallid Harrier
Circus pygargus	Monagu’s Harrier
Accipiter gentilis	Goshawk
Accipiter nisus	Sparrowhawk
Accipiter brevipes	Levant Sparrowhawk
Buteo buteo	Buzzard
Buteo rufinus	Long-legged Buzzard
Buteo lagopus	Rough-legged Buzzard
Aquila pomarina	Lesser Spotted Eagle
Aquila clanga	Spotted Eagle
Aquila nipalensis	Steppe Eagle
Aquila heliaca	Imperial Eagle
Aquila adalberti	Spanish Imperial Eagle
Aquila chrysaetos	Golden Eagle
Hieraaetus pennatus	Booted Eagle
Hieraaetus fasciatus	Bonelli’s Eagle
Pandion haliaetus	Osprey
Falco naumanni	Lesser Kestrel
Falco tinnunculus	Kestrel
Falco vespertinus	Red-footed Falcon
Falco columbarius	Merlin
Falco subbuteo	Hobby
Falco eleonorae	Eleonora’s Falcon
Falco biarmicus	Lanner
Falco cherrug	Saker Falcon
Falco rusticolus	Gyrfalcon
Falco peregrinus	Peregrine Falcon
Bonasa bonasia	Hazel Grouse
Lagopus lagopus	Willow Grouse
Lagopus mutus	Ptarmigan
Tetrao tetrix	Black Grouse
Tetrao urogallus	Capercaillie
Alectoris chukar	Chukar

Table S1 (continued)

Scientific name	**Common name** (English)
Alectoris graeca	Rock Partridge
Alectoris rufa	Red-legged Partridge
Alectoris barbara	Barbary Partridge
Perdix perdix	Grey Partridge
Coturnix coturnix	Quail
Phasianus colchicus	Pheasant
Rallus aquaticus	Water Rail
Porzana porzana	Spotted Crake
Porzana parva	Little Crake
Porzana pusilla	Baillon's Crake
Crex crex	Corncrake
Gallinula chloropus	Moorhen
Porphyrio porphyrio	Purple Gallinule
Fulica atra	Coot
Fulica cristata	Crested Coot
Grus grus	Crane
Anthropoides virgo	Demoiselle Crane
Tetrax tetrax	Little Bustard
Otis tarda	Great Bustard
Haematopus ostralegus	Oystercatcher
Himantopus himantopus	Black-winged Stilt
Recurvirostra avosetta	Avocet
Burhinus oedicnemus	Stone Curlew
Glareola pratincola	Collared Pratincole
Glareola nordmanni	Black-winged Pratincole
Charadrius dubius	Little Ringed Plover
Charadrius hiaticula	Ringed Plover
Charadrius alexandrinus	Kentish Plover
Charadrius asiaticus	Caspian Plover
Charadrius morinellus	Dotterel
Pluvialis apricaria	Golden Plover
Pluvialis squatarola	Grey Plover
Vanellus gregarius	Sociable Plover
Vanellus vanellus	Lapwing
Calidris alba	Sanderling
Calidris minuta	Little Stint
Calidris temminckii	Temminck's Stint
Calidris maritima	Purple Sandpiper
Calidris alpina	Dunlin
Limicola falcinellus	Broad-billed Sandpiper
Philomachus pugnax	Ruff
Lymnocryptes minimus	Jack Snipe
Gallinago gallinago	Snipe
Gallinago media	Great Snipe
Gallinago stenura	Pintail Snipe
Scolopax rusticola	Woodcock
Limosa limosa	Black-tailed Godwit
Limosa lapponica	Bar-tailed Godwit
Numenius phaeopus	Whimbrel
Numenius arquata	Curlew
Tringa erythropus	Spotted Redshank
Tringa totanus	Redshank
Tringa stagnatilis	Marsh Sandpiper
Tringa nebularia	Greenshank
Tringa ochropus	Green Sandpiper
Tringa glareola	Wood Sandpiper
Xenus cinereus	Terek Sandpiper

Table S1 (continued)

Scientific name	**Common name** (English)
Actitis hypoleucos	Common Sandpiper
Arenaria interpres	Turnstone
Phalaropus lobatus	Red-necked Phalarope
Phalaropus fulicarius	Grey Phalarope
Stercorarius pomarinus	Pomarine Skua
Stercorarius parasiticus	Arctic Skua
Stercorarius longicaudus	Long-tailed Skua
Stercorarius skua	Great Skua
Larus ichthyaetus	Great Black-headed Gull
Larus melanocephalus	Mediterranean Gull
Larus minutus	Little Gull
Larus ridibundus	Black-headed Gull
Larus audouinii	Audouin’s Gull
Larus canus	Common Gull
Larus fuscus	Lesser Black-backed Gull
Larus argentatus	Herring Gull
Larus cachinnans	Yellow-legged Gull
Larus hyperboreus	Glaucous Gull
Larus marinus	Great Black-backed Gull
Rissa tridactyla	Kittiwake
Pagophila eburnea	Ivory Gull
Gelochelidon nilotica	Gull-billed Tern
Sterna caspia	Caspian Tern
Sterna sandvicensis	Sandwich Tern
Sterna dougallii	Roseate Tern
Sterna hirundo	Common Tern
Sterna paradisaea	Arctic Tern
Sterna albifrons	Little Tern
Chlidonias hybridus	Whiskered Tern
Chlidonias niger	Black Tern
Chlidonias leucopterus	White-winged Black Tern
Uria aalge	Guillemot
Uria lomvia	Brünnich’s Guillemot
Alca torda	Razorbill
Cepphus grylle	Black Guillemot
Alle alle	Little Auk
Fratercula arctica	Puffin
Pterocles orientalis	Black-bellied Sandgrouse
Pterocles alchata	Pin-tailed Sandgrouse
Columba livia	Rock Dove / Feral Pigeon
Columba oenas	Stock Dove
Columba palumbus	Woodpigeon
Streptopelia decaocto	Collared Dove
Streptopelia turtur	Turtle Dove
Clamator glandarius	Great Spotted Cuckoo
Cuculus canorus	Cuckoo
Cuculus saturatus	Oriental Cuckoo
Tyto alba	Barn Owl
Otus scops	Scops Owl
Bubo bubo	Eagle Owl
Nyctea scandiaca	Snowy Owl
Surnia ulula	Hawk Owl
Glaucidium passerinum	Pygmy Owl
Athene noctua	Little Owl
Strix aluco	Tawny Owl
Strix uralensis	Ural Owl
Strix nebulosa	Great Grey Owl

Table S1 (continued)

Scientific name	**Common name** (English)
Asio otus	Long-eared Owl
Asio flammeus	Short-eared Owl
Aegolius funereus	Tengmalm’s Owl
Caprimulgus europaeus	Nightjar
Caprimulgus ruficollis	Red-necked Nightjar
Apus apus	Swift
Apus pallidus	Pallid Swift
Apus melba	Alpine Swift
Apus caffer	White-rumped Swift
Alcedo atthis	Kingfisher
Merops apiaster	Bee-eater
Coracias garrulus	Roller
Upupa epops	Hoopoe
Jynx torquila	Wryneck
Picus canus	Grey-headed Woodpecker
Picus viridis	Green Woodpecker
Dryocopus martius	Black Woodpecker
Dendrocopos major	Great Spotted Woodpecker
Dendrocopos syriacus	Syrian Woodpecker
Dendrocopos medius	Middle Spotted Woodpecker
Dendrocopos leucotos	White-backed Woodpecker
Dendrocopos minor	Lesser Spotted Woodpecker
Picoides tridactylus	Three-toed Woodpecker
Chersophilus duponti	Dupont’s Lark
Melanocorypha calandra	Calandra Lark
Calandrella brachydactyla	Short-toed Lark
Calandrella rufescens	Lesser Short-toed Lark
Galerida cristata	Crested Lark
Galerida theklae	Thekla Lark
Lullula arborea	Woodlark
Alauda arvensis	Skylark
Eremophila alpestris	Shore Lark
Riparia riparia	Sand Martin
Ptyonoprogne rupestris	Crag Martin
Hirundo rustica	Swallow
Hirundo daurica	Red-rumped Swallow
Delichon urbicum	House Martin
Anthus campestris	Tawny Pipit
Anthus berthelotii	Berthelot’s Pipit
Anthus hodgsoni	Olive-backed Pipit
Anthus trivialis	Tree Pipit
Anthus pratensis	Meadow Pipit
Anthus cervinus	Red-throated Pipit
Anthus spinoletta	Water Pipit
Anthus petrosus	Rock Pipit
Motacilla flava	Yellow Wagtail
Motacilla citreola	Citrine Wagtail
Motacilla cinerea	Grey Wagtail
Motacilla alba	White Wagtail
Bombycilla garrulus	Waxwing
Cinclus cinclus	Dipper
Troglodytes troglodytes	Wren
Prunella modularis	Dunnock
Prunella montanella	Siberian Accentor
Prunella atrogularis	Black-throated Accentor
Prunella collaris	Alpine Accentor
Cercotrichas galactotes	Rufous Bush Robin

Table S1 (continued)

Scientific name	**Common name** (English)
Erithacus rubecula	Robin
Luscinia luscinia	Thush Nightingale
Luscinia megarhynchos	Nightingale
Luscinia calliope	Siberian Rubythroat
Luscinia svecica	Bluethroat
Tarsiger cyanurus	Red-flanked Bluetail
Phoenicurus ochruros	Black Redstart
Phoenicurus phoenicurus	Redstart
Saxicola rubetra	Whinchat
Saxicola torquata	Stonechat
Oenanthe isabellina	Isabelline Wheatear
Oenanthe oenanthe	Wheatear
Oenanthe pleschanka	Pied Wheatear
Oenanthe hispanica	Black-eared Wheatear
Oenanthe leucura	Black Wheatear
Monticola saxatilis	Rock Thrush
Monticola solitarius	Blue Rock Thrush
Zoothera dauma	White's Thrush
Turdus torquatus	Ring Ouzel
Turdus merula	Blackbird
Turdus ruficollis	Black-throated Thrush
Turdus pilaris	Fieldfare
Turdus philomelos	Song Thrush
Turdus iliacus	Redwing
Turdus viscivorus	Mistle Thrush
Cettia cetti	Cetti's Warbler
Cisticola juncidis	Fan-tailed Warbler
Locustella lanceolata	Lanceolated Warbler
Locustella naevia	Grasshopper Warbler
Locustella fluviatilis	River Warbler
Locustella luscinioides	Savi's Warbler
Acrocephalus melanopogon	Moustached warbler
Acrocephalus paludicola	Aquatic Warbler
Acrocephalus schoenobaenus	Sedge warbler
Acrocephalus agricola	Paddyfeild Warbler
Acrocephalus dumetorum	Blyth's Reed Warbler
Acrocephalus palustris	Marsh Warbler
Acrocephalus scirpaceus	Reed warbler
Acrocephalus arundinaceus	Great Reed warbler
Hippolais opaca / H. pallida	Western / Eastern Olivaceous Warbler
Hippolais olivetorum	Olive-tree Warbler
Hippolais icterina	Icterine Warbler
Hippolais polyglotta	Melodious Warbler
Sylvia balearica / S. sarda	Balearic / Marmora's Warbler
Sylvia undata	Dartford Warbler
Sylvia conspicillata	Spectacled Warbler
Sylvia cantillans	Subalpine Warbler
Sylvia melanocephala	Sardinian Warbler
Sylvia rueppelli	Rüppell's Warbler
Sylvia hortensis / S. crassirostris	Western / Eastern Orphean Warbler
Sylvia nisoria	Barred Warbler
Sylvia curruca	Lesser Whitethroat
Sylvia communis	Whitethroat
Sylvia borin	Garden Warbler
Sylvia atricapilla	Blackcap
Phylloscopus trochiloides	Greenish Warbler
Phylloscopus borealis	Arctic Warbler

Table S1 (continued)

Scientific name	**Common name** (English)
Phylloscopus inornatus	Yellow-browed Warbler
Phylloscopus bonelli / P. orientalis	Bonelli's / Eastern Bonelli's Warbler
Phylloscopus sibilatrix	Wood Warbler
Phylloscopus collybita / P. ibericus	Chiffchaff / Iberian Chiffchaff
Phylloscopus trochilus	Willow Warbler
Regulus regulus	Goldcrest
Regulus ignicapillus	Firecrest
Muscicapa striata	Spotted Flycatcher
Ficedula parva	Red-breasted Flycatcher
Ficedula semitorquata	Semi-collared Flycatcher
Ficedula albicollis	Collared Flycatcher
Ficedula hypoleuca	Pied Flycatcher
Panurus biarmicus	Bearded Tit
Aegithalos caudatus	Long-tailed Tit
Parus palustris	Marsh Tit
Parus lugubris	Sombre Tit
Parus montanus	Willow Tit
Parus cinctus	Siberian Tit
Parus cristatus	Crested Tit
Parus ater	Coal Tit
Parus caeruleus	Blue Tit
Parus cyanus	Azure Tit
Parus major	Great Tit
Sitta krueperi	Krüper's Nuthatch
Sitta europaea	Nuthatch
Sitta neumayer	Rock Nuthatch
Tichodroma muraria	Wallcreeper
Certhia familiaris	Treecreeper
Certhia brachydactyla	Short-toed Treecreeper
Remiz pendulinus	Penduline Tit
Oriolus oriolus	Golden Oriole
Lanius collurio	Red-backed Shrike
Lanius minor	Lesser Grey Shrike
Lanius excubitor / L. meridionalis	Great Grey Shrike / Southern Grey Shrike
Lanius senator	Woodchat Shrike
Lanius nubicus	Masked Shrike
Garrulus glandarius	Jay
Perisoreus infaustus	Siberian Jay
Cyanopica cyanus	Azure-winged Magpie
Pica pica	Magpie
Nucifraga caryocatactes	Nutcracker
Pyrrhocorax graculus	Alpine Chough
Pyrrhocorax pyrrhocorax	Chough
Corvus monedula	Jackdaw
Corvus frugilegus	Rook
Corvus corone / C. cornix	Carrion Crow / Hooded Crow
Corvus corax	Raven
Sturnus vulgaris	Starling
Sturnus unicolor	Spotless Starling
Sturnus roseus	Rose-coloured Starling
Passer domesticus	House Sparrow
Passer × italiae	Italian Sparrow
Passer hispaniolensis	Spanish Sparrow
Passer montanus	Tree Sparrow
Petronia petronia	Rock Sparrow
Montifringilla nivalis	Snowfinch
Fringilla coelebs	Chaffinch

Table S1 (continued)

Scientific name	**Common name** (English)
Fringilla montifringilla	Brambling
Serinus serinus	Serin
Serinus citrinella	Citril Finch
Carduelis chloris	Greenfinch
Carduelis carduelis	Goldfinch
Carduelis spinus	Siskin
Carduelis cannabina	Linnet
Carduelis flavirostris	Twite
Carduelis flammea / C. cabaret	Common Redpoll / Lesser Redpoll
Carduelis hornemanni	Arctic Redpoll
Loxia leucoptera	Two-barred Crossbill
Loxia curvirostra	Crossbill
Loxia scotica	Scottish Crossbill
Loxia pytyopsittacus	Parrot Crossbill
Bucanetes githagineus	Trumpeter Finch
Carpodacus erythrinus	Scarlet Rosefinch
Pinicola enucleator	Pine Grosbeak
Pyrrhula pyrrhula	Bullfinch
Coccothraustes coccothraustes	Hawfinch
Calcarius lapponicus	Lapland Bunting
Plectrophenax nivalis	Snow Bunting
Emberiza citrinella	Yellowhammer
Emberiza cirlus	Cirl Bunting
Emberiza cia	Rock Bunting
Emberiza cineracea	Cinereous Bunting
Emberiza hortulana	Ortolan Bunting
Emberiza caesia	Cretzschmar's Bunting
Emberiza rustica	Rustic Bunting
Emberiza pusilla	Little Bunting
Emberiza aureola	Yellow-breasted Bunting
Emberiza schoeniclus	Reed Bunting
Emberiza pallasi	Pallas's Reed Bunting
Emberiza melanocephala	Black-headed Bunting
Miliaria calandra	Corn Bunting
