# Supplementary material for: Potential Impacts of Climatic Change on European Breeding Birds
Source: PLoS One. 2008 Jan 16;3(1):e1439. doi: 10.1371/journal.pone.0001439 (PMC2186378; doi:10.1371/journal.pone.0001439)
Supplement: Table S2 — Population estimates for Europe for 10 endemic and near-endemic species† and for examples of their widespread relatives. (0.02 MB PDF) [file pone.0001439.s002.pdf]

Table S2: Population estimates for Europe for 10 endemic and near-endemic species[†] and for examples of their widespread relatives

Endemic and near-endemic species with potential overlap < 10 %		Related comparator species		Ratio of European populations
**Endemics**	**Total European Population[‡]**		**Total European Population[‡]**	
Aquila adalberti Spanish Imperial Eagle	155 (150 – 160)	*Aquila chrysaetos* Golden Eagle	5412 (5239 – 5616)	34·916
Sylvia balearica / S. sarda Balearic Warbler / Marmora's Warbler	39,921 (33,317 – 49,003)	*Sylvia undata* Dartford Warbler	2,624,617 (2,025,456 – 3,635,791)	65·745
Cyanopica cyanus Azure-winged Magpie	281,423 (257,683 – 350,557)	*Pica pica* Magpie	9,028,781 (7,696,302 – 11,984,013)	32·083
Serinus citrinella Citril Finch	260,091 (251,722 – 279,620)	*Serinus serinus* Serin	11,511,967 (10,095,566 – 13,496,108)	44·261
Loxia scotica Scottish Crossbill	612 (300 – 1250)	*Loxia curvirostra* Crossbill	1,202,176 (1,004,141 – 1,632,437)	1964·340
**Near-endemics**				
Calonectris diomedea Cory's Shearwater	142,722 (121,536 – 172,591)	*Fulmarus glacialis* Fulmar	2,759,431 (2,261,216 – 3,549,427)	19·334
Puffinus yelkouan / P. mauretanicus Levantine Shearwater / Balearic Shearwater	21,241 (18,036 – 25,343)	*Puffinus puffinus* Manx Shearwater	302,342 (284,260 – 323,490)	14·234
Larus melanocephalus Mediterranean Gull	249,862 (183,925 – 339,963)	*Larus ridibundus* Black-headed Gull	2,087,768 (1,985,158 – 2,246,547)	8·356
Larus audouinii Audouin's Gull	13,355 (13,304 – 13,410)	*Larus cachinnans* Yellow-legged Gull	208,122 (198,341 – 220,323)	15·584
Acrocephalus paludicola Aquatic Warbler	4684 (2852 – 7856)	*Acrocephalus scirpaceus* Reed Warbler	3,138,033 (2,683,670 – 3,735,367)	669·947
			**Median**	**33·500**

[†] The five endemic and five near-endemic species tabulated are those with potential range overlaps of < 10 % of their present range extent for the HadCM3 SRES B2 climatic change scenario.

[‡] Population estimates are from: Hagemeijer EJM, Blair MJ, editors (1997) *The EBCC Atlas of European Breeding Birds: Their distribution and abundance*. London: T. & A.D. Poyser. 903 p.
